# Management of COVID‐19 patients in Fangcang shelter hospital: clinical practice and effectiveness analysis

**DOI:** 10.1111/crj.13293

**Published:** 2020-10-28

**Authors:** Pei Liu, Hongmei Zhang, Xiang Long, Wei Wang, Danting Zhan, Xinke Meng, Duoyun Li, Lingwei Wang, Rongchang Chen

**Affiliations:** ^1^ Department of Respiratory and Critical Care Medicine Shenzhen Key Laboratory of Respiratory Diseases Shenzhen Institute of Respiratory Diseases, Shenzhen People’s Hospital (The Second Clinical Medical College, Jinan University; The First Affiliated Hospital, South University of Science and Technology) Shenzhen China; ^2^ Emergence Department Shenzhen People’s Hospital (The Second Clinical Medical College, Jinan University; The First Affiliated Hospital, South University of Science and Technology) Shenzhen China; ^3^ Department of Respiratory and Critical Care Medicine Peking University Shenzhen Hospital Shenzhen China; ^4^ Department of Respiratory and Critical Care Medicine Zhongnan Hospital of Wuhan University Wuhan China; ^5^ Intensive Care Unit Shenzhen Second People’s Hospital Shenzhen China; ^6^ Department of Infectious Diseases Huazhong University of Science and Technology Shenzhen Union Hospital (Nanshan Hospital) Shenzhen China

**Keywords:** COVID‐19, Fangcang shelter hospital, management protocol package

## Abstract

Fangcang shelter (Cabin) hospitals were set up in order to cope with the rapid growth of confirmed cases of coronavirus disease 2019 (COVID‐19) in Wuhan, China at a time when there were insufficient beds in designated hospitals. This paper describes the layout and functioning of a typical Fangcang shelter hospital, Wuhan Dongxihu Fangcang shelter Hospital, where the author has worked, the working mechanism, experience and effectiveness. A set of patient management protocols was employed for daily practice, which included preset criteria and procedure for admission, examination, medication treatment, referral and discharge. WeChat platform with different groups was used for communication, ward round, test appointments and patient data communication. All these procedures and mechanisms of working enabled the effective management of a larger number of patients with relatively few doctors. As a result, 442 mild or moderate COVID‐19 patients in Hall C were successfully managed by a team of 40 doctors, with 246 (56%) patients were cured and discharged from the Fangcang shelter hospital while the remaining 196 (44%) patients were referred on to designated hospitals for further treatment. The reasons for referral included poor resolution in computerized tomography (CT) scan (59%), persistently positive severe acute respiratory syndrome coronavirus 2 by PCR after 9 days of admission (16%), deterioration in CT image (4%), development of dyspnoea (1%) and other (4%) or unclear reasons (16%) due to no record of reasons for referral on the document. There were no deaths and no complaints from the patients in Hall C. In summary, the Fangcang shelter hospital could be run successfully with a set of patient management protocols under conditions of limited facilities and medical staff. It was effective and safe in isolating patients, providing basic medical care and early identification of potential severe cases. This experience may provide a successful example of a working mechanism for the prevention and control of the COVID‐19 pandemic worldwide.

## 1 INTRODUCTION

Coronavirus disease 2019 (COVID‐19) has become a great threat to the health of all human beings.^1^, ^2^, ^3^, ^4^ The pandemic started in Wuhan China in the late December 2019 and reached its peak with more than 3000 new cases in the middle of February 2020, resulting in a serious shortage of beds, medical personnel and protective equipment in designated hospitals in Wuhan at that time.^5^ Despite many efforts and strategies implemented by the government, there was public dissatisfaction with the management and quarantine facilities available at that time. In response, a Chinese expert panel proposed to build simple temporary hospitals, named Fangcang shelter or Cabin hospitals, with the aim of providing sufficient beds and accommodation for all confirmed patients with COVID‐19. Within a period of 1 month or more, 16 Fangcang shelter hospitals were built in Wuhan, accommodating approximately 12 000 mild and moderate patients. This was a more reliable strategy to isolate patients and prevent further transmission.^6^ However, Fangcang shelter hospitals are simple temporary institutions without standard medical hospital facilities and a full complement of medical professionals. Thus, a large number of patients had to be managed by a relatively small number of medical staff using an operative mechanism adapted to the acute situation. The author has worked in a typical Fangcang shelter hospital, Wuhan Dongxihu Fangcang shelter Hospital. He now describes the layout of this hospital, its working mechanism and its effectiveness from his own personal experience.

## 2 MATERIALS AND METHODS

### 2.1 The structure of Dongxihu Fangcang shelter hospital

**FIGURE 1:**
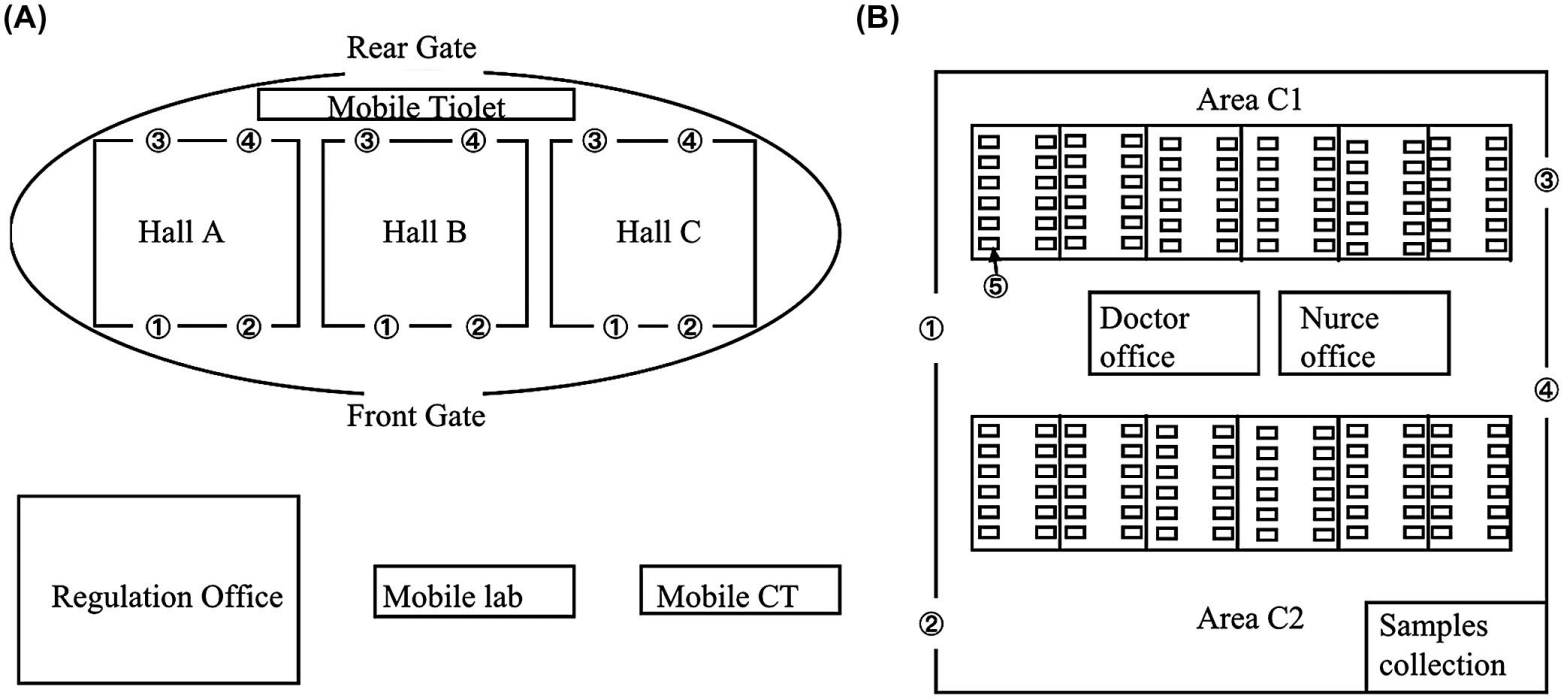
The floor plan of Fangcang shelter hospital. (A) The plan of Fangcang shelter hospital. (B) The floor plan of Hall C. ① Medical staff entrance, ② medical staff exit, ③ patient access, ④ medical waste outlet, ⑤ ward beds

Generally, the Fangcang shelter hospital has been built in large spaces with adequate ventilation, and in locations that can be easily sealed for free access.^6^ Dongxihu Fangcang shelter hospital is located in the Wuhan Cultural Exhibition Centre, as shown in Figure 1A. The hospital is divided into Hall A, Hall B and Hall C, with 1400 beds. Nearby, there are buildings that are used as headquarters and that shelters a mobile laboratory and CT room. Hall C has 450 beds, and is divided into Area C1 and Area C2. Each area is divided into several units composed of 30‐40 beds. Doctor office and Nurse office are located in the centre of Hall C. Samples collection room is located in the corner of Hall C, which is convenient for disinfection in a relatively closed environment. The entrance for patients, staff and waste exit are all relatively separated. At each access, there were staff responsible for infection control to regulate the entrance or exit and for helping the medical staff in wearing and taking off personal protective equipment. The floor plan of Hall C is shown in Figure 1B.

### 2.2 Medical management processes

There were 42 doctors from 26 hospitals working in Hall C, including 11 respiratory doctors, 6 critical care doctors, 4 infection doctors and 21 doctors from other departments. Two doctors worked in the regulation office and the other 40 doctors were divided into eight groups with five doctors in each group. Each group had one head doctor responsible for medical quality control, decision‐making and communication with regulation office and headquarters. So, one doctor would be responsible for 10 to 15 patients.

#### 2.2.1 Shift duty inside the ward room

The ward room was isolated inside the Fangcang shelter hospital. At any time during the 24 hours of the day, there were five doctors inside the ward room to supervise and care for all patients. Eight groups of doctors in rotation entered the ward room for a shift of 6 hours. So, each group entered the ward room once every 2 days. Inside the ward room, one doctor could cover 100 to 120 patients. During the ward round, doctors spent around 3 minutes on average with each patient. The main task was to check the patient face‐to‐face and identify any sign of disease progression. In case of deteriorating of the patient’s condition, the head of the group re‐evaluated the patient again before contacting the regulation office and headquarters for referring the patient onwards to designated hospitals. The head doctor was also responsible for the verification of patients who met the criteria for discharge.

#### 2.2.2 Duty outside the ward room

**FIGURE 2:**
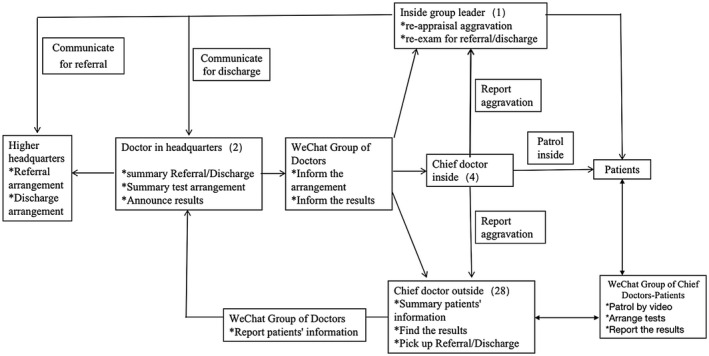
The tasks and duties of each doctor

The tasks and duties of each doctor is shown in Figure 2. Outside of the ward room, each doctor was responsible for the management of 10 to 15 patients. Each doctor and his/her patients under his/her responsibility were connected within a WeChat group and supervision of the patients occurred through WeChat video‐chats. If the patient did not have the WeChat application, the doctor would contact the patient with his/her cell phone. The appointment for specific tests or examination, such as CT and PCR, was also implemented via WeChat. So, even if the responsible doctor was off‐duty out of the ward room, his/her patients could be supervised through the WeChat platform. The communication and collaboration between the responsible doctors and doctors on‐duty in the ward room were also important aspects in the management of patients. The collaboration between doctors is illustrated in Figure 2.

### 2.3 Management protocol package employed in the Fangcang shelter hospital

In order to improve the efficiency and standardise the management inside the Fangcang shelter hospital, protocol package including admission criteria, management and medications, as well as referral criteria and discharge criteria were established. The protocol package was as follows:

#### 2.3.1 Admission criteria

Generally, only the confirmed cases with mild to moderate severity should be accommodated. Details of criteria are as following:

1. Patients with mild or moderate severity;
2. Patients with ability to look after themselves independently, and can walk without support;
3. Patients without other diseases such as severe organ dysfunction;
4. Patients without history of mental illness;
5. In the resting state, the peripheral figure blood oxygen saturation without supplementary oxygen a (SpO2) >93%, and a respiratory rate(RR) <30 times/min.

#### 2.3.2 Evaluation and treatment protocol

##### Evaluation flow chart

1. Vital signs recording: body temperature, breathing frequency (RR), heart rate (HR), blood pressure (BP) and SpO2 recorded four times daily at 08:00, 12:00, 16:00 and 20:00.
2. Chest CT scan: before admission and 1 week after admission. If the lung lesions did not improve, CT scan would be repeated every 5 days until resolved or referral criteria met;
3. PCR test for SARS2‐coV: before admission and 1 week after admission, and then, every 2 days until the yield was negative for at least two consecutive times.

##### Treatment protocol

1. Antiviral treatment: Arbidol and COVID‐19 Granules (extract of Chinese herb medicine) were used according to the guideline issued by National Health Commission of PR China^7^, ^8^, ^9^:

Arbidol: 0.2 g there times per day for 5 days;

COVID‐19 Granules: two bags, two times per day, until discharge.

- Antimicrobial drug: use one of the two drugs for 5‐7 days.

Moxifloxacin (18 age or above): 0.4 g once, once per day, oral;

Azithromycin: 0.5 g once, once per day, oral.

- Symptomatic treatment:

If the patient has a fever, physical cooling is recommended; if the body temperature >38.5°C, Ibuprofen Capsules (300 mg once) would be used. For patient with cough, Compound Methoxyphenamine Capsules would be used.

#### 2.3.3 Discharge criteria

In general, the subject could be discharged if disease has been obviously alleviated and the PCR was negative for at least twice.^7^, ^8^, ^9^ The preset criteria were as following:

1. The body temperature is normal for more than 3 days;
2. Respiratory symptoms almost disappear;
3. CT scan shows that the lung lesions are improving obviously;
4. The PCR was negative for at least two consecutive times (the sampling interval is at least 1 day);
5. Sp02 >93% without supplementary oxygen;
6. The whole course of the disease has exceeded 14 days.

#### 2.3.4 Criteria and processes for referral to designated hospitals

In general, once the patients deteriorated and met the criteria of severe case or whose medical condition could not be managed in the Fangcang shelter hospital, they would be referred to designated hospitals. The criteria and processes for referral are as follows:

##### Preset referral criteria

1. Respiratory distress with RR ≥ 30 BPM;
2. SpO2 ≤ 93% while breathing room air at rest;
3. Respiratory failure and requiring ventilator support;
4. Shock;
5. Multiple organ failure and requiring ICU monitoring and treatment;
6. CT imaging shows that the lung lesions have progressed to more than 50% in 24‐48 hours;
7. Patients over 60 years old with uncontrolled serious chronic diseases, including hypertension, diabetes, coronary heart disease, tumour, COPD and immunosuppressive population, etc.

##### Additional referral criteria during the real‐life practice

In order to enhance the safety of the patients, some additional referral criteria were employed during the real‐life practice. These additional referral criteria are as follows:

1. The chest CT showing progression after 1 week admission into the Fangcang shelter hospital;
2. The chest CT showing poor improvement after 12 days since admission;
3. PCR SARS2‐cov persistent positive after 9 days since admission;
4. Persistent high fever with temperature ≥38.5°C for more than 3 days since admission.

### 2.4 Data analysis

We observed the number of patients who were cured or referred. At the same time, we analysed the reason for referring. The data are presented as the mean and standard deviation. Analyses was performed by Statistical Product and Service Solutions (SPSS) 25.

## 3 RESULTS

### 3.1 General information of patients in Hall C

From 9 February 2020 to 8 March 2020, 442 patients were admitted into Hall C. Average age was 50.0 ± 13.4 years. There were 224 males and 218 females.

### 3.2 The outcome of patients in Hall C

**FIGURE 3:**
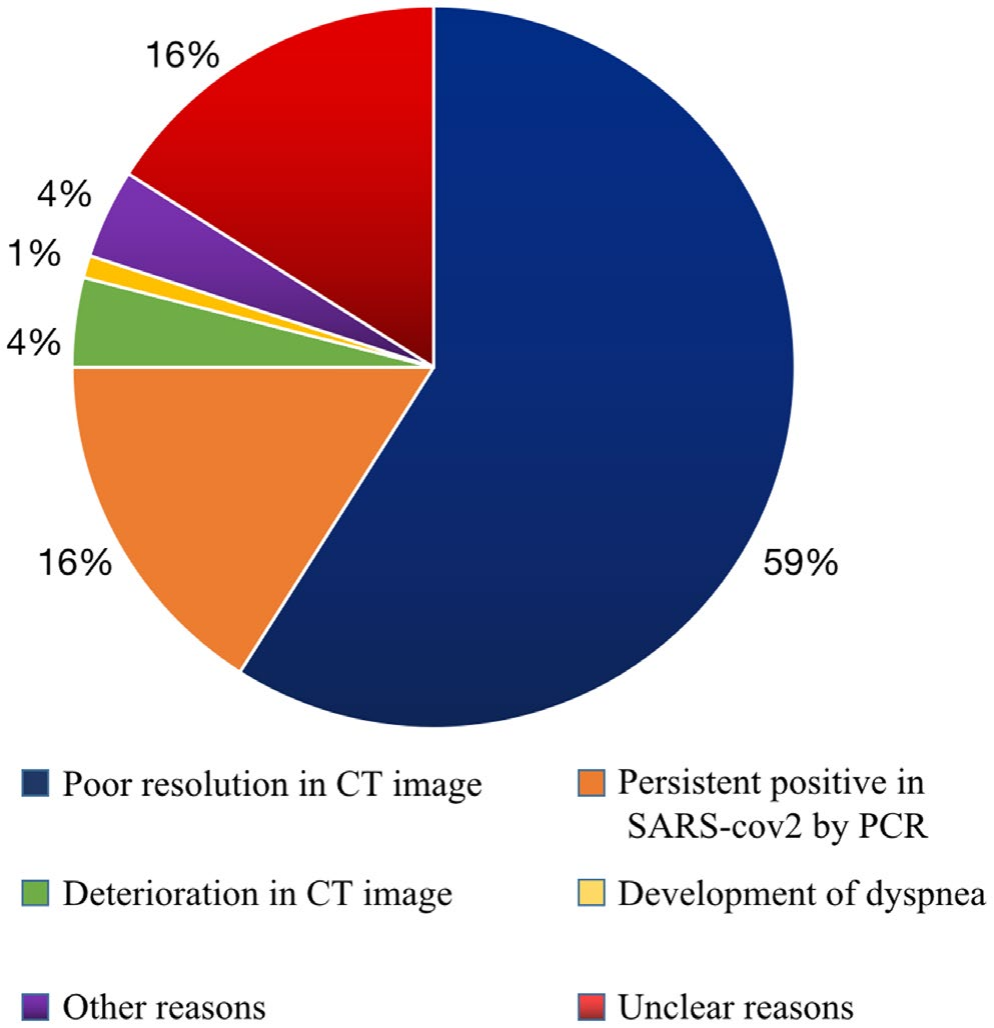
The clinical outcome of Hall C

The clinical outcome of patients in Hall C is shown in Figure 3. About 246 (56%) patients were cured and discharged from the Fangcang shelter hospital. About 196 (44%) patients were referred to designated hospital for further treatment. The causes of referral included poor improvement in CT image (59%), persistent PCR SARS2‐cov positive (16%), progression of lung lesions on chest CT (4%), development of dyspnoea (1%) and other (4%) or unclear reasons (16%) due to no record of reasons for referral on the document. There were no deaths as well as no complaints received from the patients in Hall C.

### 3.3 Infection and accidents of medical staffs in Hall C

During the operative period from 9 February 2020 to 8 March 2020, there were zero medical staff infection of COVID‐19 and no medical accidents in the Dongxihu Fangcang shelter hospital.

## 4 DISCUSSION

Fangcang shelter hospitals were initiated to use in 5 February 2020 and completely closed on 10 March 2020. It had provided more than 12 000 beds for accommodation and isolation of mild and ordinary COVID‐19 patients. It have been proven to be an efficient and safe strategy with the majority of the patients completely recovered with no infection transmitted to the medical staff and with no medical incidents as well as no complaints received from patients.^6^ In Hall C of Dongxihu Fangcang shelter hospitals, we employed the management protocol package, which allowed us to manage a large number of patients with relatively small number of medical staff.

### 4.1 The necessity for management protocol package in Fangcang shelter hospitals

Fangcang shelter hospitals were established temporarily in response to the urgent situation of increasing numbers of COVID‐19 positive patients. There were several important factors that were confronted. First, medical staff worked in it were from different places outside Wuhan and with different specialty background. Second, the work load was pretty heavy due to low doctor to patient ratio. Doctors on duty inside the ward room were responsible for management of 100‐120 patients, which greatly exceeded the service limit of the doctors and made it impossible to work in a normal usual routine schedule. Third, wearing personal protection equipment impaired the efficiency of the doctor’s work. Hence the necessity of adopting a management protocol package in these Fangcang shelter hospitals.

### 4.2 Management protocol package could reduce the workload of doctors working in Fangcang shelter hospitals

Before the management protocol package was adopted, the doctors not on duty outside the ward room could not be involved in the care of the patients. So, all the medical care of the more than 400 patients was provided by the five doctors on duty inside the ward room of Fangcang shelter hospital, with a schedule that included ward rounds, monitoring of patients, making appointment for specific tests or examination, writing medical documents and evaluating patients who met the criteria of discharge or referral. After the management protocol package was established, the efficiency of work was increased and doctors outside the ward room could work through the WeChat platform and follow personally the progress of the patients under their care on a daily basis. This dramatically reduced the burden of the doctors working inside the ward room.

### 4.3 Possibility of management of patients via WeChat group‐chat

Patients who were admitted by Fangcang shelter hospitals were all mild and moderate. The proportion of the patients with progressing chest CT presentation, development of dyspneoa and other presentations that exceeded the competence of Fangcang shelter hospitals was only 9% (these patients were severe or might develop to severe). Besides, the progression of COVID‐19 patients were identified mainly by objective parameters such as heart rate, respiratory rate, CT scan and Sp02, which can be easily communicated by WeChat groups. Thus, the WeChat‐based ward round was feasible under these special and unusual conditions.

### 4.4 Benefits of managing patients via WeChat group

After the doctors and patients were connected by WeChat groups, the patients developed a strong sense of belonging to a group and got to know their responsible doctors, which significantly alleviated the stress of patients and greatly reducing the need for having group events. Besides, the WeChat groups reduced the need of doctors to see the patients face‐to‐face, which was key to reducing the risk of infection and use of personal protection equipment which was in short supply. With the WeChat platform, every doctor could be involved in the management of patients even when they are not on duty inside the ward room, which led to a reduction in the workload of the doctors on duty inside the ward room.

### 4.5 The criteria for referral to designated hospitals

The criteria for referral to designated hospitals should be considered in order to reduce the burden of designated hospitals and risk of severe patients staying in Fangcang hospitals. At the beginning, there were shortage of ward beds in designated hospitals. So, the original preset criteria for referral were more stringent. At a later stage when the ward bed in the designated hospitals became more available, less stringent criteria were used that reduce the risk of potentially severe cases being kept in Fangcang hospitals. We felt that the criteria for referral should be adjusted according to the situation, in order to make a good balance between available ward beds in designated hospitals and the safety of the patients.

### 4.6 Limitations

As the Fangcang shelter hospitals were set up in response to the new emergence of COVID‐19 pandemic, there was no previous experience to refer to. There were some limitations in the workings of our Fangcang hospital. For instance, the hospital information system, laboratory information system, picture archiving & communication system and other information management systems were not available. So, we had to rely on WeChat platform. Web‐based remote operation system was not available. With the 5G technology, it is possible to establish remote operation system with majority of the medical observation and documents related work being carried out outside the ward room. There are many aspects that could be improved using state‐of‐the‐art technology in the situation of an infectious disease pandemic, which will enable us to cope better with the disease management and disease control. Furthermore, this is a retrospective analysis. In 16% of referral cases, we failed to identify the true reasons for referral due to no record on the document. We also failed to trace the final outcomes of those transferred to designated hospitals.

### 4.7 Conclusions

In summary, the Fangcang shelter hospital was run successfully with patient management protocol package in situation of limited facilities and medical staff. It was effective and safe in isolating patients, providing basic medical care and identified very early on of potential severe cases. WeChat platform was successfully used to supervise and communicate with COVID‐19 patients, which minimised the medical staff’s direct contact with patients and avoiding transmission. The experience of Hall C of Wuhan Dongxihu Fangcang shelter Hospital provides a successful example of a working mechanism for the prevention and control of the COVID‐19 pandemic worldwide.

## ETHICS APPROVAL

The study was a retrospective study, and the study protocol was approved by the ethics committee of Zhejiang University.

## CONFLICT OF INTEREST

No potential conflicts of interest were disclosed.

## AUTHOR CONTRIBUTIONS

All authors approved the final version.

*Conceived and revised this article:* Chen, Wang

*Participated in the treatment of patients in the Fangcang shelter hospital, acquired key information and wrote this article:* Liu

*Processed and visualised the data, and reviewed the literature:* Chen, Liu

*Worked in the Fangcang shelter hospital and provided data:* Long, Wang

*Wrote and translated the article:* Zhang

*Wrote the first draft:* Zhan

*Worked in the Fangcang shelter hospital and contributed to the advice of the management protocol package:* Meng, Li

## Data Availability

Data sharing is not applicable to this article as no data sets were generated or analysed during the current study.
